# Supplementary material for: Comparison of seven SNP calling pipelines for the next-generation sequencing data of chickens
Source: PLoS One. 2022 Jan 31;17(1):e0262574. doi: 10.1371/journal.pone.0262574 (PMC8803190; doi:10.1371/journal.pone.0262574)
Supplement: S1 Word — (DOCX) [file pone.0262574.s009.docx]

**SNP calling pipelines for chicken NGS sets**

**1. Qualitative Control with Trimmomatic (version 0.39)**

java -jar trimmomatic-0.39.jar PE -threads 16 Sample_1.clean.fq Sample_2.clean.fq Sample_forward_paired.fq Sample_forward_unpaired.fq Sample_reverse_paired.fq Sample_reverse_unpaired.fq ILLUMINACLIP:TruSeq3-PE-2.fa:2:30:10 LEADING:3 TRAILING:3 SLIDINGWINDOW:4:15 CROP:135 MINLEN:135

**2. Alignment with bowtie 2 (version 2.2.9)**

bowtie2-build ref.fa Gallus_gallus-5.0

bowtie2 -p 8 --reorder -x Gallus_gallus-5.0 -X 500 -1 prefix_1.fq -2 prefix_2.fq -S prefix.sam >& prefix.sam.log

samtools sort -o prefix.bam -@ 8 prefix.sam

samtools index prefix.bam

**3. Seven SNP calling pipelines**

**3.1 Freebayes** (version: freeBayes v1.1.0-60-gc15b070-dirty)

freebayes -f ref.fa aln.bam >var.vcf

vcftools --vcf var.vcf --remove-indels --recode --recode-INFO-all --out prefix.snp

**3.2 16GT** (version 1.0)

# Build reference index

soap3-dp-builder ref.fa

BGS-Build ref.fa.index

# Convert bam to snapshot

bam2snapshot -i ref.fa.index -b prefix.bam -o output/prefix

# Call variants

snapshotSnpcaller -i ref.fa.index -o output/prefix

perl txt2vcf.pl output/prefix.txt sampleName ref.fa > prefix.variants.vcf

vcftools --vcf prefix.variants.vcf --remove-indels --recode --recode-INFO-all --out prefix.snp

**3.3 Bcftools-single (bcftools version 1.6)**

bcftools mpileup -Ou -f ref.fa prefix.bam | bcftools call -Ov -mv -o prefix.var.vcf

vcftools --vcf prefix.var.vcf --remove-indels --recode --recode-INFO-all --out prefix.snp

**3.4 Bcftools-multiple (bcftools version 1.6)**

bcftools mpileup -Ou -f ref.fa prefix1.bam prefix2.bam ... prefixn.bam | bcftools call -Ov -mv -o prefix.multi.var.vcf

vcftools --vcf prefix.multi.var.vcf --remove-indels --recode --recode-INFO-all --out prefix.multi.snp

**3.5 Varscan2-single (****Varscan version 2.4.3)**

samtools mpileup -f ref.fa prefix.bam > prefix.sam.mpileup

java -jar ~/VarScan.v2.4.3.jar mpileup2cns prefix.sam.mpileup --output-vcf 1 --variants > prefix.vcf

vcftools --vcf prefix.vcf --remove-indels --recode --recode-INFO-all --out prefix.snp

**3.6 Varscan2-multiple (Varscan version 2.4.3)**

samtools mpileup -f ref.fa prefix1.bam prefix2.bam ... prefixn.bam | java -jar ~/VarScan.v2.4.3.jar mpileup2cns --output-vcf 1 --variants > prefix.multi.vcf

vcftools --vcf prefix.multi.vcf --remove-indels --recode --recode-INFO-all --out prefix.multi.snp

**3.7 GATK (version 4.2.3.0)**

**##To create known snp and indel vcf files**

wget <ftp://ftp.ensembl.org/pub/release-94/variation/vcf/gallus_gallus/gallus_gallus.vcf.gz>

java -jar ~/picard.jar CreateSequenceDictionary REFERENCE=ref.fa OUTPUT= ref.dict

vcftools --vcf gallus_gallus.vcf --keep-only-indels --recode --recode-INFO-all --out indel

java -jar ~/picard.jar SortVcf I= indel.recode.vcf O=**indel.sorted.vcf** SD=ref.dict

vcftools --vcf gallus_gallus.vcf --remove-indels --recode --recode-INFO-all --out snp

java -jar ~/picard.jar SortVcf I=snp.recode.vcf O=**snp.sorted.vcf** SD=ref.dict

java -jar ~/picard.jar SortVcf I= gallus_gallus.vcf O= **gallus_gallus.sorted.vcf** SD=ref.dict

**##Add RG information and resort order**

gatk AddOrReplaceReadGroups -I prefix.bam -O prefix_rg_added_sorted.bam -SO coordinate -RGID prefix.bam -RGLB library -RGPL illumina -RGPU machine -RGSM prefix

**##Markduplication**

gatk MarkDuplicates -I prefix_rg_added_sorted.bam -O prefix_dedupped.bam -CREATE_INDEX true -VALIDATION_STRINGENCY SILENT -M prefix_output.metrics

**##Recalibrate base quality scores**

gatk BaseRecalibrator -R ref.fa -I prefix_dedupped.bam --known-sites snp.sorted.vcf --known-sites indel.sorted.vcf -O prefix_recal_data.table

gatk ApplyBQSR -R ref.fa -I prefix_dedupped.bam -bqsr prefix_recal_data.table -O prefix_recal_reads.bam

**##Single sample GVCF calling on DNAseq**

gatk HaplotypeCaller -R ref.fa -I prefix_recal_reads.bam -ERC GVCF --dbsnp gallus_gallus.sorted.vcf -stand-call-conf 30 -O prefix.raw.g.vcf

**##Joint Genotyping using GenotypeGVCFs**

gatk GenomicsDBImport --variant prefix1.raw.g.vcf --variant prefix2.raw.g.vcf ... --variant prefixn.raw.g.vcf --genomicsdb-workspace-path ./gatkdb -L 1 -L 2 -L 3 -L 4 -L 5 -L 6 -L 7 -L 8 -L 9 -L 10 -L 11 -L 12 -L 13 -L 14 -L 15 -L 16 -L 17 -L 18 -L 19 -L 20 -L 21 -L 22 -L 23 -L 24 -L 25 -L 26 -L 27 -L 28 -L 30 -L 31 -L 32 -L 33 -L W -L Z -L MT -L LGE64

gatk GenotypeGVCFs -R ref.fa -V gendb://gatkdb -O prefix.vcf --tmp-dir=./tmp

vcftools --vcf prefix.vcf --remove-indels --recode --recode-INFO-all --out prefix.snp
